# Voice in Female Singers Affected by Gynæcological Disorders

**Published:** 1891-07

**Authors:** Carl. H. Von Klein

**Affiliations:** Dayton, O.


### SELECTIONS.

### Voice in Female Singers Affected by Gynecological Disorders.

BY CARL. H. VON KLEIN, A.M., M.D., OF DAYTON, O.

I am requested by a well-known ovariotomist to state my observation of the voices of female singers when affected by gynecological disorders, hence the following is a brief statement of my observation.

The most difficult cases the laryngologist has to contend with are diseases of the throat caused by disturbance of the ovaries. It is a common thing to meet with cases of acute inflammation of the tonsils, larynx, pharynx and fauces in females during their menstrual period. I have observed the voice in many professional choir singers who have applied to me for treatment during the menstrual period, defective in gravity, force and timbre, producing in many cases a husky sound, as of a low, masculine order.

A laryngologist of acute hearing who will train his ear to the recognition of sounds and acquaint himself with a known voice, can detect a menstruate nine times out of ten. It is a known fact that all prima donnas try to avoid engagements during their expected period. It is a recognized fact from time immemorial that extirpation of the testicles will greatly change the voice in males. Unto this day the operation is practiced in some parts of the civilized world.

The finest male chorus I ever heard was by a band of eunuchs at the Alexandre Nefsky Church at St. Petersburg, Russia, who were prepared for that purpose. Born eunuchs, or hermaphrodites, generally have voices of feminine order, but do not make good singers on account of their sluggishness and want of animal propensities. It is said in order to make a good singer one must be in love. It is undisputable that impediment in the male organs influences the male voice, so, too, impediment in female organs influences the female voice.

In many cases of ovarian disturbance, enlargement and hypertrophy of the tonsils and of the soft palate are observed, hence the laryngologist oftentimes can accomplish but very little without the assistance of a competent gynecologist.

